# Filamin C: a novel component of the KCNE2 interactome during hypoxia

**DOI:** 10.5830/CVJA-2015-049

**Published:** 2016

**Authors:** Annika Neethling, Jomien Mouton, Valerie Corfield, Carin de Villiers, Craig Kinnear, Ben Loos

**Affiliations:** DST /NRF Centre of Excellence in Biomedical Tuberculosis Research, SA MRC Centre for Tuberculosis Research, Division of Molecular Biology and Human Genetics, Department of Biomedical Sciences, Faculty of Medicine and Health Sciences, Stellenbosch University, South Africa; DST /NRF Centre of Excellence in Biomedical Tuberculosis Research, SA MRC Centre for Tuberculosis Research, Division of Molecular Biology and Human Genetics, Department of Biomedical Sciences, Faculty of Medicine and Health Sciences, Stellenbosch University, South Africa; DST /NRF Centre of Excellence in Biomedical Tuberculosis Research, SA MRC Centre for Tuberculosis Research, Division of Molecular Biology and Human Genetics, Department of Biomedical Sciences, Faculty of Medicine and Health Sciences, Stellenbosch University, South Africa; DST /NRF Centre of Excellence in Biomedical Tuberculosis Research, SA MRC Centre for Tuberculosis Research, Division of Molecular Biology and Human Genetics, Department of Biomedical Sciences, Faculty of Medicine and Health Sciences, Stellenbosch University, South Africa; DST /NRF Centre of Excellence in Biomedical Tuberculosis Research, SA MRC Centre for Tuberculosis Research, Division of Molecular Biology and Human Genetics, Department of Biomedical Sciences, Faculty of Medicine and Health Sciences, Stellenbosch University, South Africa; Department of Physiological Sciences, Faculty of Science, Stellenbosch University, Stellenbosch, South Africa

**Keywords:** LQTS, KCNE2, filamin C (FLNC), hypoxia, arrhythmia

## Abstract

**Aim:**

KCNE2 encodes for the potassium voltage-gated channel, KCNE2. Mutations in KCNE2 have been associated with long-QT syndrome (LQTS). While KCNE2 has been extensively studied, the functions of its C-terminal domain remain inadequately described. Here, we aimed to elucidate the functions of this domain by identifying its protein interactors using yeast two-hybrid analysis.

**Methods:**

The C-terminal domain of KCNE2 was used as bait to screen a human cardiac cDNA library for putative interacting proteins. Co-localisation and co-immunoprecipitation analyses were used for verification.

**Results:**

Filamin C (FLNC) was identified as a putative interactor with KCNE2. FLNC and KCNE2 co-localised within the cell, however, a physical interaction was only observed under hypoxic conditions.

**Conclusion:**

The identification of FLNC as a novel KCNE2 ligand not only enhances current understanding of ion channel function and regulation, but also provides valuable information about possible pathways likely to be involved in LQTS pathogenesis.

## Aim

Long-QT syndrome (LQTS) is a cardiac repolarisation disorder with an estimated global prevalence of 1:2 000 to 1:7 000.^1^,^2^ It is characterised by a prolonged QT interval on a surface electrocardiogram (ECG), with symptoms including syncope, cardiac arrest and sudden death.^1^,^3^,^4^ Occasionally, sudden cardiac death may be the first and only manifestation of LQTS.^5^,^6^

To date, different types of LQTS (LQT1–LQT13), classified according to the primary disease causal gene, have been identified, with more than 700 mutations leading to disease pathogenesis.^7^,^8^ Yet a large number of patients with clinically diagnosed LQTS have no mutations within any of the known LQTS causal genes,^9^-^11^ and numerous patients, despite carrying the same disease-causing mutation, display variable phenotypic expression and disease penetrance.^12^ To complicate matters further, LQTS can also be acquired through the use of certain prescribed medications, such as antipsychotics, antidepressants and antibiotics,^13^,^14^ adding to the growing challenge of clinical management and treatment of affected individuals.

The LQT type 6 (LQT6) causal gene, *KCNE2* encoding for the potassium voltage-gated channel subfamily E member 2 (KCNE2) protein,^15^ has been implicated in the development of inherited, acquired and sporadic forms of LQTS.^13^,^16^-^18^ This protein consists of an extracellular N-terminal, a transmembrane and intracellular C-terminal domain. It comprises the beta-(β) subunits of ion channel complexes and co-assembles with many different alpha- (α) subunits, including the frequently studied human *Ether-à-go-go*-related (HERG) channel protein encoded for by the potassium voltage-gated channel, subfamily H (eag-related), member 2 (*KCNH2*) gene.^15^,^17^,^19^ In combination with *KCNE2*, properties of the different ion channel currents are modulated,^20^ assisting in cardiac pacemaker activity and repolarisation to ensure adequate myocardial recharging and the maintenance of a regular rhythm.^15^,^21^-^23^

A unique quality of many cardiac ion channels, including those containing KCNE2 and HERG, is their ability to adapt to hypoxic conditions. Hypoxia, defined as the decrease in available oxygen, causes changes in the electrical characteristics of ion channels and has been reported to predispose individuals to fatal arrhythmias.^24^-^27^ Additionally, hypoxic conditions affect the expression, folding, maturation and trafficking of various channels.^28^-^30^ In a recent study, it was noted that the expression of genes from the *KCNE* family (including *KCNE2*) could be affected by hypoxia in the heart.^31^ It has been observed that acute ischaemic hearts of rats after myocardial infarction show increased expression of KCNE proteins, attributable to hypoxia.^31^

The intricacy of processes causing and modifying cardiac arrhythmias highlights the importance of identifying the protein macromolecular complexes and pathways involved. Taking into consideration the relevance of *KCNE2* in the context of ion channel regulation and LQTS, this study aimed to identify interactors with this β-subunit; specifically focusing on its cytoplasmic C-terminal domain, for which functional roles remain inadequately described.

Using yeast two-hybrid analysis, we identified filamin C (FLNC) as a KCNE2-interacting protein. FLNC and its paralogs, filamin A (FLNA) and filamin B (FLNB), act as scaffolding proteins and have been implicated in a number of cellular stress responses,^32^-^38^ including several hypoxia-related effects.^35^–^38^ For this reason, co-localisation and co-immunoprecipitation (Co-IP) analyses for verification of this interaction were conducted both under normoxic and hypoxic conditions.

Here, we show that, under normoxic and hypoxic conditions, FLNC and KCNE2c co-localised within the cell. However, FLNC and KCNE2 only co-immunoprecipitated under hypoxic conditions, suggesting that while these two proteins are located in close proximity to one another within the cell, it is only under conditions of cellular stress that a physical interaction between the two exists. The data presented here provide evidence to suggest that *KCNE2* may play a role in hypoxia-induced arrhythmias.

## Methods

## KCNE2 construct

A fragment encoding the C-terminal of *KCNE2* gene (amino acid 72-123) was amplified from human genomic DNA by means of polymerase chain reaction (PCR). The PCR reaction employed *KCNE2* C-terminal-specific primers with two restriction enzyme sites (*Nde*I and *Eco*R1) (Table 1) for subsequent cloning into the CLONTECH yeast two-hybrid (Y2H) bait vector, pGBKT7 (pGBKT7-*KCNE2*), in-frame with the *GAL4*-DNA binding domain (*GAL4*BD). The integrity of the sequence and the conservation of the *GAL4* domain reading frame of the resulting construct were verified via sequencing.

**Table 1:** Nucleotide sequences of primers used to amplify the C-terminal of KCNE2

*Primer*	*Sequence (5’-3’)*	*Ta (°C)*
KCNE2-forward^Nde1^	5’ - ACTGCAGAACATATGCTCAAATCCAAGAGACGG - 3’	50
KCNE2-reverse^EcoR1^	5’ - ACTGCAGAAGAATTCCTATCAGGGGAACATTTTGAAC - 3’	51

°C: degrees Celsius; Ta: annealing temperature; KCNE2: potassium voltagegated ion-channel subfamily E member 2. The bold text represents a tag, which facilitates restriction enzyme digestion, while the underlined sequences correspond to the Nde1 and EcoR1 restriction enzyme sites, respectively. The short italic sequence (CTA) symbolises the stop codon, and the remaining text represents the sequence of the primer, which will anneal to the DNA in the PCR amplification reaction.

## Yeast two-hybrid (Y2H) library screen

The *Saccharomyces cerevisiae* strain, AH109 (BD Biosciences, Clontech, USA), was transformed with the pGBKT7-*KCNE2* construct and mated with the *S cerevisiae strain*, Y187, which was pre-transformed with a MATCHMAKER human cardiac cDNA library (BD Biosciences, Clontech, USA). Subsequently, the library screen was conducted according to manufacturer’s recommendations.

The prey plasmids, from colonies expressing the three essential reporter genes (*HIS3, ADE2* and *MEL1*), were isolated from the diploid yeast cells and were retransformed into *S cerevisiae* strain Y187 to analyse their ability to activate the reporter genes when mated with heterologous baits (Table 2). Prey peptides showing specific interaction with the *KCNE2* C-terminal domain were sequenced and the in-frame open reading frame (ORF) sequences were analysed using BLASTN and BLASTP against public databases (http://ncbi.nlm.nih.gov/blast).

**Table 2:** S cerevisiae bait strains

*S cerevisiae bait strains*	*Plasmid type*
AH109 pGBKT7-KCNE2	Positive control plasmid
AH109 pGBKT7	Non-recombinant plasmid
AH109 pGBKT-53*	Control bait plasmid
AH109 pGBKT7-WFS1	Negative control plasmid

*The pGBKT7 vector containing the human p53 gene. *KCNE2*: potassium voltage-gated ion-channel subfamily E member 2; *WFS1*: Wolfram syndrome 1.

## Cell culture

The H9C2 rat-derived cardiac myoblasts (American Typer Culture Collection, USA) were grown in Dulbecco’s modified Eagle medium (DMEM, Lonza, CHE) containing 10% foetal bovine serum (FBS, Biochrom, GER) and 1% penicillin/streptomycin (Pen/Strep, Biochrom, GER) until they reached 80% confluency. For co-localisation, 10 000 cells were seeded onto glass cover slips in each well of a six-well plate (8-cm^2^ culture dishes) and incubated until 80% confluency was reached, while for Co-IP, cells were grown in 175-cm2 flasks until they reached 80% confluency. Differentiation medium (DMEM containing 1% horse serum and 1% Pen/Strep) was subsequently added to each well of the six-well plate and the 175-cm^2^ flasks. Cells were differentiated for 10–14 days.

For hypoxia induction, the differentiation medium was removed and replaced with Esumi buffer (138.6 mM NaCl, 12 mM KCl, 1 mM MgCl_2_, 1 mM CaCl_2_.H_2_O, and 4 mM Hepes, pH 6.2).^39^ Culture dishes and flasks were then placed in a chamber where a hypoxic environment was created by flushing the system with a 1% O_2_ gas mixture at a flow rate of 20 l/min, for approximately four minutes. The cells were then incubated in the hypoxic chamber at 37°C for two hours.

For Co-IP experiments, 5 ml of pre-warmed trypsin was used to detach the cells from the growth surface of the flasks. The cells were then centrifuged at 4ºC for three minutes at 2 500 rpm. The supernatant was discarded and the pellet resuspended in 1 ml of phosphate-buffered saline (PBS) and re-pelleted at 9 000 rpm for two minutes. The PBS was removed and the cells were then lysed with ice-cold lysis buffer (50 mM Hepes, 5 M NaCl, 0.5 M EDTA, 1% Triton X-100, 1 M Na_3_VO_4_) containing protease inhibitor cocktail tablets [one tablet EDTA-free protease inhibitor cocktail tablet per 20 ml lysis buffer and 1 mM phenylmethylsulfonylfluoride (PMSF) (Sigma-Aldrich, USA)].

Approximately 0.5 ml of ZROB05 Ceria zirconium oxide beads (0.5 mm diameter) (Next Advance Inc, USA) was added to the suspension and it was placed in a Bullet blender® (Gentaur, GBR) for one minute. The blending step was repeated three times at five-minute intervals. The cells were then pelleted by centrifugation at 9 000 rpm for two minutes, after which the supernatant was collected. A Bradford assay was used for protein concentration determination,^40^ to ensure equivalent amounts of protein per sample were subjected to sodium dodecyl sulphatepolyacrylamide gel electrophoresis (SDS-PAGE) analysis.

## Co-localisation

For co-localisation experiments, the differentiation media and Esumi buffer was removed from the differentiated H9C2 rat-derived cardiomyocytes on the glass cover slips and the cells were rinsed with PBS. The cells were permeabilised with methanol for five minutes at –20°C and fixed with 4% paraformaldehyde for five minutes at room temperature. The cells were then washed three times with PBS for 10 minutes and blocked in 1% BSA for one hour at room temperature. Following the blocking step, the cells were again washed three times with PBS for 10 minutes and incubated at 4°C overnight with rabbit anti-KCNE2 (Abcam, Biocom Biotech, RSA, 1:50) and goat anti-FLNC (Santa Cruz Biotechnology Inc, USA, 1:50) primary antibodies diluted in 1% BSA.

The cells were then washed three times with PBS for 10 minutes and stained with Alexa 488 donkey anti-rabbit (Jackson ImmunoResearch Laboratories Inc, USA, 1:500) and Cy3 donkey anti-goat (Jackson ImmunoResearch Laboratories Inc, USA, 1:500) secondary antibodies in PBS for 90 minutes in the dark at room temperature. Afterwards, the cells were washed three times with PBS for 10 minutes, and Hoechst H-33342 was added for nuclear staining [Sigma-Aldrich (Pty) Ltd, RSA, 1:200; 10 mg/ml], followed by a 10-minute incubation at room temperature.

Subsequently, the cover slips with the stained cells were mounted onto glass slides using Mowiol (Jackson ImmunoResearch Laboratories Inc, USA) containing n-propylgallate as the anti-fade reagent and kept at 4°C in the dark until viewing. Samples were acquired using the Carl Zeiss Confocal LSM 780 Elyra S1, equipped with a LSM780 GaAsP detector, using a Plan Apochromat 63×/1.4 Oil DIC M27 or an alpha Plan- Apochromat 100×/1.46 oil DIC objective (Central analytical facility, Cell Imaging Unit, Stellenbosch University, RSA). The samples were excited with a 488-nm and 561-nm laser underutilisation of a MBS 488/561 beam splitter.

Images were acquired through z-stacking with an increment of 0.3-μm step width, and projected as maximum-intensity projections using ZEN software (black edition, 2011). Thresholds were determined using appropriate control images acquired for cells individually stained (single-stain) for KCNE2 and FLNC, respectively. The background was adjusted for all acquired images using images of cells only stained with secondary control antibodies.

## Co-immunoprecipitation

Cells were harvested and the lysates were pre-cleared with protein G agarose beads (KPL Inc, USA) for 30 minutes at 4°C. The pre-cleared lysates (150 μg/total protein) were incubated with 1 μg of either rabbit polyclonal anti-KCNE2 (Santa Cruz Biotechnology Inc, USA) or goat polyclonal anti-FLNC (Santa Cruz Biotechnology Inc, USA) antibody rotating overnight at 4°C.

To capture the protein complexes, 60 μl of protein G agarose beads were added to the lysate and incubated for an additional hour rotating at 4°C. The complexes were then washed three times, each time removing the supernatant after centrifugation and adding fresh lysis buffer that contained protease inhibitors and PMSF. Proteins were eluted by addition of 1× SDS-PAGE sample buffer [95% Laemmli sample buffer (Bio-Rad Laboratories Inc, USA), 5% β-mercapto-ethanol], denatured for five minutes at 95°C and separated using 4–15% SDS-PAGE gels for Western blot analysis. Two negative controls, a non-relevant antibody control (HA-probe; Santa Cruz Biotechnology Inc, USA) and a protein G agarose control (without antibody) were included in all Co-IP experiments

## Western blot analysis

Following co-IP, proteins were separated on 4–15% SDS-PAGE gels and transferred to a polyvinylidene difluoride (PVDF) membrane (Thermo Scientific, USA) by means of the iBlot® system (Invitrogen, USA). Membranes were blocked with 5% fat-free powdered milk, supplemented with Tris-buffered saline Tween-20 (TBST, 0.01% Tween-20), for one hour at room temperature. Membranes were then incubated at 4°C overnight with the appropriate primary antibodies (Santa Cruz Biotechnology Inc, USA, 1:200 anti-KCNE2; 1:1 000 anti-FLNC), diluted with 5% milk in TBST.

Subsequently, the membranes were washed with TBST and incubated for one hour at room temperature with the corresponding horseradish peroxidase (HRP) conjugated secondary antibodies (Santa Cruz Biotechnology Inc, USA, 1:2 000 donkey anti-rabbit; 1:2 000 donkey anti-goat), diluted with 5% milk in TBST. Following incubation with the secondary antibody, the membranes were washed for 30 minutes at room temperature.

The SuperSignal® West Pico chemiluminescence substrate kit (Thermo Scientific, USA) was then used according to the manufacturer’s instructions and the membranes were exposed for two minutes to CL-Xposure™ autoradiography film (Thermo Scientific, USA). The autoradiography film was developed using an Amersham hyperprocessor automatic autoradiography film processor (Amersham Pharmacia Biotech UK Ltd, UK) prior to final analyses.

## Results

## FLNC as a novel interactor with KCNE2 under hypoxic conditions

The Y2H screen identified FLNC (GenBank: NP_001449.3) as a KCNE2 (GenBank: NP_751951.1) interactor with the binding regions located between amino acids 2637–2725 of FLNC and amino acids 72–123 of KCNE2. These 88 amino acids of FLNC are positioned at the end of the C-terminal domain, shown to be involved in self-dimerisation.^32^

**Fig. 1.**
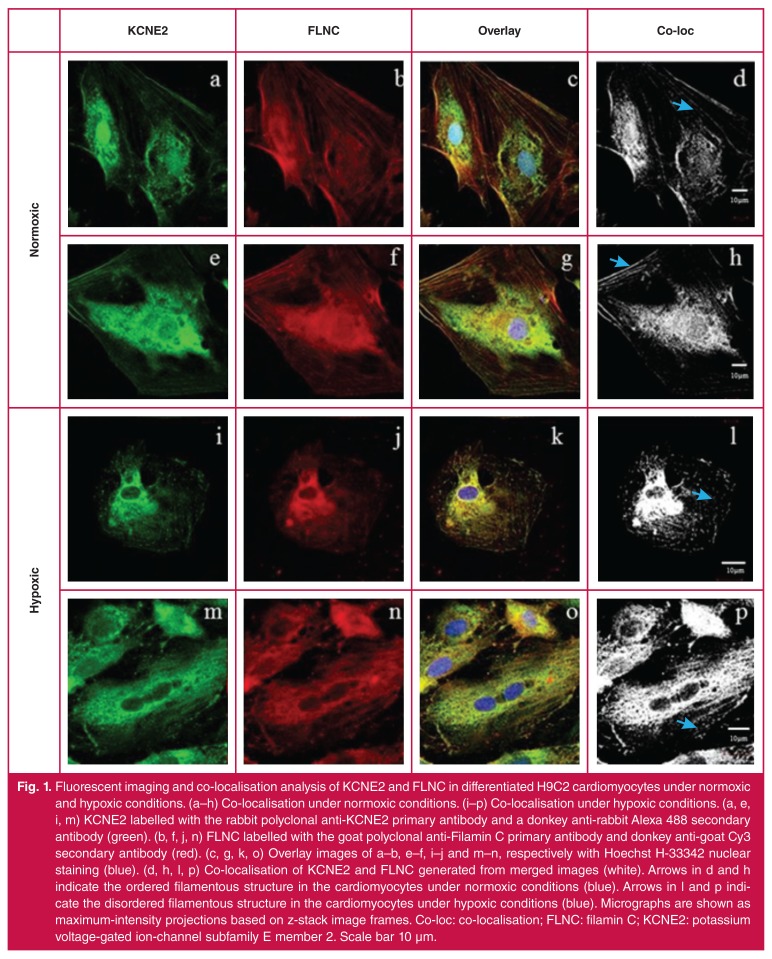
Fluorescent imaging and co-localisation analysis of KCNE2 and FLNC in differentiated H9C2 cardiomyocytes under normoxic and hypoxic conditions. (a–h) Co-localisation under normoxic conditions. (i–p) Co-localisation under hypoxic conditions. (a, e, i, m) KCNE2 labelled with the rabbit polyclonal anti-KCNE2 primary antibody and a donkey anti-rabbit Alexa 488 secondary antibody (green). (b, f, j, n) FLNC labelled with the goat polyclonal anti-Filamin C primary antibody and donkey anti-goat Cy3 secondary antibody (red). (c, g, k, o) Overlay images of a–b, e–f, i–j and m–n, respectively with Hoechst H-33342 nuclear staining (blue). (d, h, l, p) Co-localisation of KCNE2 and FLNC generated from merged images (white). Arrows in d and h indicate the ordered filamentous structure in the cardiomyocytes under normoxic conditions (blue). Arrows in l and p indicate the disordered filamentous structure in the cardiomyocytes under hypoxic conditions (blue). Micrographs are shown as maximum-intensity projections based on z-stack image frames. Co-loc: co-localisation; FLNC: filamin C; KCNE2: potassium voltage-gated ion-channel subfamily E member 2. Scale bar 10 μm.

Imaging analysis revealed a strong co-localisation signal between KCNE2 and FLNC at the cell membrane, filamentous structures and the cytoplasm of differentiated H9C2 rat-derived cardiomyocytes under normoxic conditions (Fig. 1a-h), while the co-localisation of these two proteins was mainly restricted to the cytoplasm under conditions of hypoxia (Fig. 1i-p). When the cells were subjected to hypoxic stress, the pattern of co-localisation, relative to that during normoxia, changed considerably at the plasma membrane (Fig. 1i-p), where decreased co-localisation of these proteins was observed. Following hypoxic stress, the internal cellular structure became disrupted and the filaments and cytoskeleton showed clear signs of disarray (Fig. 1i-p). The less well-defined signal appearance of co-localisation seen in Fig. 1l and p may be attributable to this disarray.

While co-localisation analysis provided convincing evidence that KNCE2 and FLNC are located in close proximity to one another within the cell, this does not necessarily mean that they physically interact. For this reason, Co-IP analysis was used to determine whether a physical interaction exists between the two.

**Fig. 2.**
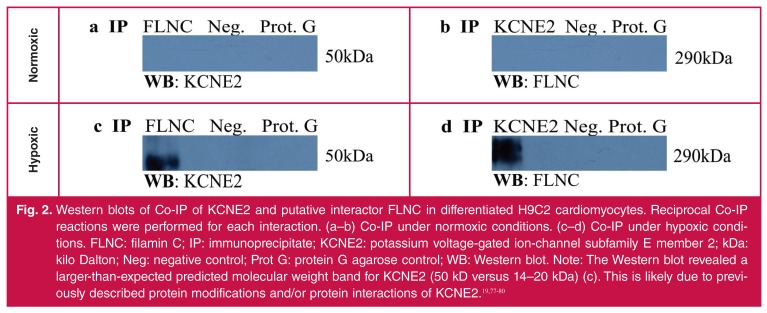
Western blots of Co-IP of KCNE2 and putative interactor FLNC in differentiated H9C2 cardiomyocytes. Reciprocal Co-IP reactions were performed for each interaction. (a–b) Co-IP under normoxic conditions. (c–d) Co-IP under hypoxic conditions. FLNC: filamin C; IP: immunoprecipitate; KCNE2: potassium voltage-gated ion-channel subfamily E member 2; kDa: kilo Dalton; Neg: negative control; Prot G: protein G agarose control; WB: Western blot. Note: The Western blot revealed a larger-than-expected predicted molecular weight band for KCNE2 (50 kD versus 14–20 kDa) (c). This is likely due to previously described protein modifications and/or protein interactions of KCNE2.^19^,^77^-^80^

Under normoxic conditions, reciprocal Co-IP experiments failed to show any physical interaction between KCNE2 and FLNC (Fig. 2c, d). However, during hypoxia an interaction between KCNE2 and FLNC was observed (Fig. 2c, d). These findings suggest that the induction of stress is essential to the interaction and one could speculate that hypoxia-induced conformational changes of FLNC are necessary for the KCNE2–FLNC interaction.

## Discussion

This study identified a novel protein–protein interaction between the cytoplasmic C-terminal domain of KCNE2 and FLNC during conditions of acute hypoxia. To date, the intracellular C-terminal domain residues of KCNE2 have been implicated in modulating HERG current density,^13^,^41^ current deactivation rates,^41^ and phosphorylation-dependant channel degradation.^19^ However, studies elaborating on specific regulatory roles for this domain remain scarce, highlighting the importance of the current findings.

The interactor identified in this study, FLNC, is located in the cytoplasm at the Z-line of the sarcomere and functions in the cytoskeleton, where it is involved in crosslinking actin filaments into networks and anchoring membrane proteins.^32^,^42^ This filamin and its main paralogs, FLNA and FLNB, act as scaffolding proteins and have been implicated in a number of cellular stress responses,^32^-^34^,^43^-^46^ including several hypoxia-related effects.^33^,^34^,^45^,^46^ FLNC specifically, is predominantly expressed in muscle tissue and is associated with cardiac abnormalities such as desminopathy, characterised by muscle weakness, conduction blocks, arrhythmias and chronic heart failure, frequently resulting in sudden cardiac death.^35^,^36^

Filamins also play an important part in cell signalling by disrupting existing interactions or by the introduction of novel interactions.^32^,^37^,^38^,^47^ Interestingly, there are several reports detailing interactions of filamin family members with ion channel subunits.^48^-^50^ Particularly noteworthy is a previously descibed association in neuronal tissue between FLNC and the potassium voltage-gated channel subfamily D member 2 (KCND2),^48^ the α-subunit of the Kv4.2 channel. That study proposed that FLNC mediates the direct link between KCND2 and the actin cytoskeleton and showed that this interaction is essential for the generation of appropriate current densities.^48^

Both neuronal and cardiac tissue contain voltage-gated ion channels responsible for controlling the excitability of neurons and cardiomyocytes. These channels allow for communication between cells in these tissues.^51^-^53^ Furthermore, a KCNE2– KCND2 interaction has been described, implicating KCNE2 in the regulation of the rapidly inactivating KCND2 α-subunit.^54^,^55^

A common theme in the observation of the ion channel interactions with filamin is the ability of filamin to influence membrane localisation.^48^-^50^ For FLNC, this process has also been shown to involve other actin-binding and auxiliary ion channel proteins.^56^

In the present study, the C-terminal of FLNC, specifically amino acids 2637–2725 (GenBank: NP_001449.3), bound to the cytoplasmic C-terminal domain of KCNE2, exclusively during conditions of hypoxic stress. This finding is consistent with a number of other studies, indicating that the C-terminal region of filamins is involved in protein interactions.^57^ The FLNC amino acid residues defined to interact with KCNE2 in this investigation correspond to a domain that is responsible for protein dimer formation and is important for actin filament bundling and cross-linking activities.^58^,^59^

The introduction of hypoxic stress is known to have profound effects on the cell.^60^ These include the disruption of ionic homeostasis, mitochondrial dysfunction resulting in impaired ATP production, induction of cell death by apoptosis or necrosis, and the generation of reactive oxygen species (ROS).^61^ Excess ROS leads to cardiac cell damage and post-ischaemic contractile dysfunction by attacking virtually all cellular components.^62^ This results in the degradation of intracellular proteins, rupture of cellular membranes (including the sarcolemma), as well as intracellular calcium ion overload,^63^ however, it has been show that cells remain viable even after extended periods of hypoxic stress.^64^ In addition to this, the actin cytoskeleton is also severely compromised and may therefore be a driving force for novel interactions. Additionally, Kesner *et al*. indicated that stressinduced conformational changes in filamins could have a direct effect on existing interactions or may influence the presence of novel interactions.^47^

Co-localisation analysis revealed that KCNE2 and FLNC co-localise under both normoxic and hypoxic conditions. However, no physical interaction could be confirmed between these two proteins during normoxic conditions using Co-IP assays. Therefore, these findings suggest that the induction of stress is essential to the interaction. Given that hypoxic stress compromises the integrity of the cellular membranes and that FLNC has also been shown to interact with the actin cytoskeleton, it is tempting to speculate that during this time of cellular stress, the interaction between FLNC and KNCE2 may be an attempt by the cell to restore cellular membrane integrity, thereby promoting cellular survival during these conditions.

If this is the case, one could further speculate that mutations in the genes encoding for KCNE2 or FLNC, or both, which weaken or abrogate their interaction could result in the cell being unable to restore membrane integrity, which could lead to acute myocardial ischaemic arrhythmogenesis. Furthermore, it may be that hypoxia-induced conformational changes of FLNC are necessary for the novel KCNE2–FLNC interaction.

During hypoxia, the pattern of co-localisation changed and the differentiated H9C2 cardiomyocytes showed signs of internal structural disruption. Both KCNE2 and FLNC displayed reduced localisation at the surface membrane (Fig. 1i, j, m, n), while the intracellular co-localisation signal was intensified. Hypoxic conditions are known to initiate extensive variations in gene expression, alter protein sub-cellular localisation, and cause the attenuation of membrane protein translation.^65^-^67^

Furthermore, these findings are consistent with reports that the HERG α-subunit, known to bind KCNE2,^16^ showed reduced membrane localisation during hypoxia.^30^,^68^ The reason for the observed decrease of these proteins at the membrane requires further investigation; however, it is interesting to note that both KCNE2 and filamins have been implicated in processes involving the internalisation of membrane proteins.^19^,^69^,^70^ Additionally, given the evidence of filamins aiding channel localisation,^69^,^70^ and the increase in *KCNE2* gene expression during hypoxia,^31^ it would be intriguing to investigate if this interaction serves a compensatory role to try to restore internally localised channels to the membrane.

The role of cytoskeletal components, including actin-binding proteins, in ion channel function and regulatory processes is a rapidly expanding field of study. Evidence supports their significant contribution towards channel trafficking and activity at the plasma membrane itself.^71^-^73^ Furthermore, there are numerous studies linking the dysfunction of cytoskeletal proteins with conduction defects and arrhythmias.^72^,^73^

This study is the first to identify the cytoskeletal protein, FLNC, as a constituent of an ion channel macromolecular complex, specifically forming part of the KCNE2 interactome. This observation was only valid during conditions of hypoxia, although it remains to be seen if other stimuli can elicit the same association. Together, FLNC and KCNE2 most likely modulate KCNE2-containing channels, especially pertaining to their surface expression.

## Conclusion

It has long been known that all cells have the ability to adapt and respond to hypoxic conditions in order to prevent the harmful effects of oxygen deprivation.74 In cardiac cells, potassium channels play a central role in this adaptation. However, inadequate adaptive responses may lead to serious cellular damage and cardiac arrhythmias. This has previously been shown in the cardiovascular system where lack of oxygen contributes to cardiac arrhythmia.^75^,^76^

This study identified and validated FLNC as an interactor with KCNE2 under conditions of hypoxia. This finding points towards new insight and understanding into the mechanisms in which KCNE2 functions, and could contribute to our understanding of the interactome in cardiovascular conditions such as LQTS. Through identification of novel KCNE2 interacting proteins, new genes can be included in searches for causal and modifying effects of novel arrhythmia disorders. These findings ultimately advocate intriguing possibilities that might lead to new therapeutic avenues being discovered.
